# The difference between clinical significance and statistical significance: an important distinction for clinical research

**DOI:** 10.55730/1300-0144.5925

**Published:** 2024-11-03

**Authors:** SADİ ELASAN

**Affiliations:** Department of Biostatistics, Faculty of Medicine, Van Yüzüncü Yıl University, Van, Turkiye

**Dear Editor**,

When evaluating the results of clinical trials, it seems little attention is paid to the difference between clinical significance and statistical significance. These two concepts are critical in interpreting research results and translating them into clinical practice. This distinction is important in medical decision-making, particularly in determining the most effective and appropriate treatments.

Statistical significance is usually determined by the p-value and evaluates whether the results obtained in testing a hypothesis are due to chance. Clinical significance assesses whether the finding makes a real difference in treatment or practice and is not the same as statistical significance [1].

A statistically significant effect (p < 0.05, etc.) only indicates that the finding is not due to chance. However, more detailed and extensive studies are needed to determine whether the effect is clinically significant [2]. For example, a finding that a drug reduces blood pressure by an average of 3.5 mmHg may be statistically significant, but long-term follow-up of patients and studies of other effects of the drug are needed to assess whether this reduction is clinically significant. Clinical significance evaluates whether the treatment or intervention has a positive and meaningful effect on real-life patient outcomes.

Clinical significance can be calculated using different methods, such as the effect size, minimal clinically important difference (MCID), response rate, risk reduction, quality of life measures, and mortality/morbidity rates [3]. For example, a treatment with a Cohen’s d value of 0.7 is considered to have a moderate effect, while a cancer treatment that prolongs life by an average of 3 months could make a significant clinical difference for patients.

In clinical research, focusing on statistical significance alone can sometimes be misleading for clinical practice [4]. For example, when working with a large sample size, even small but clinically insignificant differences may be statistically significant. In a clinical trial with 10,000 participants, a weight loss of 0.5 kg in the treatment group may be statistically significant but not clinically significant.

In summary, researchers and clinicians need to consider both statistical and clinical significance when evaluating results. Clinical significance is measured by the effect of the change achieved after treatment on patients’ quality of life, morbidity, or mortality. I believe that paying more attention to the concepts of statistical and clinical significance in the interpretation and reporting of clinical research results will increase the contribution of research to real-world applications. Therefore, it is important to provide further expert guidance so that the scientific community can better understand and account for the difference between these two concepts.
